# Gene signature associated with resistance to fluvastatin chemoprevention for breast cancer

**DOI:** 10.1186/s12885-022-09353-2

**Published:** 2022-03-17

**Authors:** Anjana Bhardwaj, Matthew D. Embury, Zhenlin Ju, Jing Wang, Isabelle Bedrosian

**Affiliations:** 1 Breast Surgical Oncology, MD Anderson Cancer Center, Houston, TX USA; 2 Department of Bioinformatics and Computational Biology, MD Anderson Cancer Center, Houston, TX USA

**Keywords:** Breast cancer prevention, Statin, Cholesterol biosynthesis, Statin response, Resistance gene signature

## Abstract

**Background:**

Although targeting of the cholesterol pathway by statins prevents breast cancer development in mouse models, efficacy is not absolute. Therefore, the goal of this study is to investigate if the upregulation in the cholesterol biosynthesis pathway genes associates with response to statin chemoprevention and may potentially be used as response biomarkers.

**Methods:**

Expression of cholesterol biosynthesis pathway genes was initially derived from the RNA sequencing of MCF10A cell line- based breast cancer progression model system and subsequently validated by quantitative PCR assay. Response to fluvastatin was assessed in vitro using the MCF10A cell line model system, including a statin resistant cell line that was generated (MCF10.AT1-R), and measured using colony forming assays. In vivo efficacy of statin for chemoprevention was assessed in the SV40C3 TAg mouse model. Mammary tumors were identified by histologic analysis of the mammary glands. Mammary glands without histologic evidence of high-grade lesions (in situ and/or invasive carcinoma) were considered responsive to statin treatment.

**Results:**

We found more than 70% of a published multi-gene fluvastatin resistance signature to be significantly upregulated during breast cancer progression and inversely correlated with statin inhibition of cellular growth and proliferation. This inherent statin resistance gene signature was also largely shared with the signature of acquired resistance to fluvastatin in MCF10.AT1-R cell line model of acquired statin resistance. These inherent resistance genes and genes exclusive to acquired statin resistance map to steroid-, and terpenoid backbone- biosynthesis pathway. We found upregulation of ~ 80% of cholesterol biosynthesis pathway genes in the tumor bearing mammary glands of SV40 C3TAg transgenic mouse model of TNBC, suggesting the involvement of cholesterol biosynthesis pathway in resistance to statin chemoprevention in vivo. A panel of 13-genes from the pathway significantly associated with response to statin treatment, as did the expression level of HMGCR alone in a mouse model of breast cancer suggesting their utility to predict the efficacy of statin chemoprevention.

**Conclusions:**

High basal level, or restorative upregulation, in the cholesterol biosynthesis pathway genes appear to be strongly associated with resistance to statin chemoprevention for breast cancer and may serve as a biomarker to tailor statin treatment to individuals who are most likely to benefit.

**Supplementary Information:**

The online version contains supplementary material available at 10.1186/s12885-022-09353-2.

## Background

Statins are widely prescribed cholesterol lowering drugs that are well tolerated and relatively inexpensive. There has been a long-standing interest in repurposing statins for prevention of breast cancer. However, data from observational studies are mixed and definitive prospective clinical trials are lacking [1–4]. A few pilot studies have tested the efficacy of statins for the prevention of breast cancer in patients, but these have offered limited insight due to use of non-informative biomarker endpoints, testing in a short preoperative window investigating anti-proliferative effects in breast cancers, or poor patient selection criteria [1–4]. In preclinical studies however, by using mouse models of triple negative breast cancer, we and others have previously shown that statins prevent breast cancer by delaying tumor onset, reducing tumor incidence and tumor burden, inhibition of DCIS lesions and extending survival [5–8]. Although these preclinical studies (including our own) are highly suggestive that statin have chemopreventive effect, they also demonstrate that statin chemoprevention is not complete. This limited statin efficacy may explain in part the lack of clear epidemiologic signal for statin use for prevention [5–8]. Hence, there is a need to better understand the mechanisms of resistance to statins and develop biomarkers of response which could help guide patient selection in order to effectively translate preclinical findings to populations that are most likely to benefit.

Preclinical data from statin use in cancer therapeutic context suggest that there are multiple factors that influence their efficacy such cancer site, subtype of cancer, genetic makeup and the degree of compensatory activation of cholesterol pathway genes that determine statin sensitivity in breast tumors. In a recent study, Kimbung et al. [2, 9] investigated the potential of statin as treatment for breast cancer. In this window trial, 25 patients with invasive primary breast cancer were administered 2 weeks of statin prior to surgery and the reduction proliferation index as measured by ki-67 in the tumor from pre- to post-treatment was measured as a surrogate for response. Expression of the genes in the cholesterol biosynthesis pathway was measured prior to treatment and the association between the baseline expression level of these genes and decrease in ki-67 investigated. The authors reported a significant association between higher baseline expression of cholesterol biosynthesis pathway genes and no reduction in ki-67 following statin treatment suggesting lack of response. These data strongly suggested that cholesterol pathway genes can serve as biomarkers of response to statin treatment and may help select cancer patients who may benefit.

In the current manuscript, we build on these findings from the breast cancer setting to study their relevance in preneoplastic state in order to determine how to best tailor the use of statins for prevention of breast cancer. The multi-step, histologic progression from normal breast epithelium to invasive breast cancer in patients is well characterized and thought to occur over many years and is accompanied by a constantly evolving molecular landscape [10]. The molecular context permissive to statin efficacy likely evolves during this multi-step progression and thus the optimal time for effective prevention of breast cancer using statins would similarly evolve. However, little is known about the optimal time for intervening with statins in high risk women to abrogate the progression to invasive breast cancer. In this manuscript we tested the hypotheses that i) histologic states further along in progression to invasive carcinoma are characterized by upregulation of cholesterol biosynthesis pathway genes and thus are more inherently resistant to statins and ii) acquired resistance to statin treatment associates with increased compensatory upregulation of a panel of cholesterol biosynthesis genes. We fulfilled these objectives by testing the relevance of compensatory activation of cholesterol biosynthesis pathway genes to breast tumorigenesis and statin efficacy by using several models (cell line and mouse model) that mimic the multi-step progression to invasive cancer.

## Methods

### Cell lines

MCF10A isogenic cell line- panel developed by Fred Miller [11, 12] that recapitulates a series of human breast cancer progression states including normal like- MCF10A (P), preneoplastic- MCF10.AT1, ductal carcinoma in situ—MCF10.DCIS and invasive breast cancer- MCF10.CA1D were used to determine the expression of a statin response and resistance gene signature at the baseline. Cell lines were obtained from Karmanos Cancer Center and DCIS.com and used with in first 10 passages. The cell lines were grown in an antibiotic free DMEM: F12 (50:50) media containing 5% horse serum, CaCl2 (Sigma Aldrich), EGF, hydrocortisone, insulin as described before [6]. All the cell culture media additives were obtained from ThermoFisher unless mentioned otherwise.

### Generation of fluvastatin resistant stable cell line

Fluvastatin sensitive preneoplastic cell line, MCF10.AT1, was exposed to gradually increasing doses of fluvastatin (from 1 µM to 20 µM) and a resistant cell line- MCF10.AT-R that has 4X the IC50 (8.5 µM) of its parental sensitive counterpart MCF10.AT1 (IC50 2.1 µM) was selected as described before [6].

### Colony formation assay

Efficacy of fluvastatin (SelleckChem), a hydrophilic statin, to inhibit the growth and survival of MCF10.AT1 and MCF10.DCIS cells was measured by colony formation assay which evaluates the ability of single cells to form colonies when plated at a low cell density of about 40–80 cells/ 6 well plate. About sixteen hours after plating the cells, these were treated with various doses of fluvastatin (5 µM and 10 µM) and allowed the cells to grow for 12 days. At the end of treatment period, culture media was aspirated, the colonies were stained with crystal violet (Sigma Aldrich) and any cell cluster bigger than 40 cells was counted manually.

### RNA extraction, gene expression- and pathway- analysis

Total cellular RNA from the cell lines and homogenized tissue samples was extracted by using Trizol as per manufacturers’ instructions as described before [13]. Next generation RNA sequencing that was performed on the total cellular RNA of MCF10A panel cell lines in our prior study [6, 14, 15] was mined to evaluate the expression pattern of a panel of gene signature of statin resistance / response. To identify the signatures of acquired resistance to fluvastatin, the total cellular RNA from 3 replicates of MCF10.AT1 and MCF10.AT1-R cells were subjected to Clariom D RNA profiling (ThermoFisher). The raw signal intensity data in the CEL files was preprocessed and normalized by RMA (Robust Multichip Average) method using Bioconductor packages “affy” and “oligo”. After preprocessing, we performed data quality assessment. The data was cleaned by removing the low expression and invariant genes. The sample A3 was removed as an outlier due to systematic variation that was revealed by a principal component analysis [16] and could not be explained biologically (suppl Fig. 2). To identify significant genes, we applied linear model for microarray data analysis [17] to compare the gene expression levels between sensitive and resistant samples. A false discovery rate (FDR) of < 5% was used to select 913 significantly deregulated genes. Pathway analysis was performed by using KEGG pathways that were downloaded from Molecular Signatures Database (MSigDB) curated by Broad Institute (http://www.gsea-msigdb.org/gsea/msigdb/index.jsp.). The expressions of pathway’s member genes were summed up as activity score for each pathway, and LIMMA was performed to compare activity scores between sensitive and resistant samples. Twenty pathways are significantly associated with the sensitivity (FDR < 5%). Most of the pathways were upregulated in resistant samples.

### Mouse studies

SV40 C3TAg mice that spontaneously develop basal like breast cancer and progress to invasive cancer by the age of 16 weeks were treated with fluvastatin (10 mg/kg/day) or vehicle control starting at the age of 5–6 weeks. The treatments continued until the age of 22 weeks at which time the animals were euthanized. At time of euthanasia, all the mouse mammary glands (cervical, thoracic and inguinal) were explanted regardless of the presence/absence of visible tumors and were processed for histology, RNA extraction, and qPCR validation of gene signatures of fluvastatin resistance. Fluvastatin efficacy was calculated by histologically grading the formalin fixed paraffin embedded mammary glands. Histological grading (on a scale of 0–5 that ranges from normal to invasive breast cancer) on the formalin fixed paraffin embedded tissues were performed as described previously [6]. Glands with a low histological grade (0–3 that range from normal to hyperplastic lesion) were considered to be non-tumor bearing and were thus considered statin responders. Mammary glands with histological grade 4 or 5 representing DCIS or invasive breast cancer were considered to be tumor bearing and thus non- responders to statin.

### Statistical analysis

All statistical and bioinformatics analyses were performed using R version 3.6.2 (https://www.r-project.org/). The rate of type I errors due to multiple comparisons was adjusted by controlling the false discovery rate (FDR) [18]. FDR of < 5% was used as significance cutoff whenever it was needed. To display the deregulation patterns of the significant genes and pathways, unsupervised hierarchical clustering heatmap was used, for which correlation was used as distance metric and ward was used as clustering method.

## Results

### Cholesterol biosynthesis pathway genes correlate with inherent resistance to fluvastatin during breast cancer progression

**Fig. 1:**
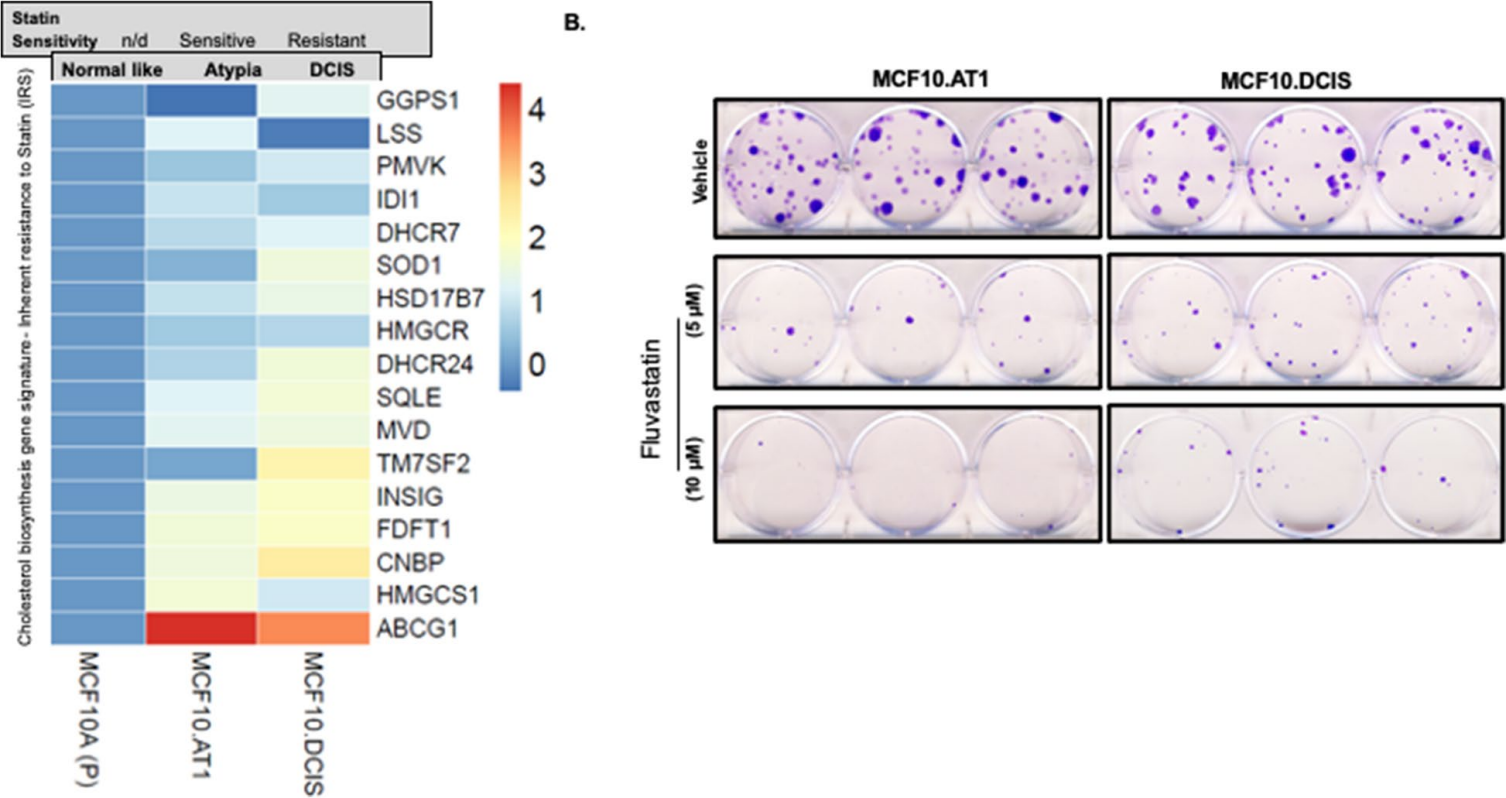
Inherent resistance to fluvastatin correlates with high baseline expression of cholesterol biosynthesis genes in a model of breast cancer progression. **A** Heatmap showing upregulation of a 17 gene panel that was derived by taking log 2 transformed folds of the genes in this supervised heatmap. The X-axis shows breast cancer progression and the Y-axis shows the gene names that were sorted by the averaged folds (top to bottom: smaller to larger folds). **B** Colony formation assay (CFA) showing the crystal violet stained colonies in the fluvastatin sensitive MCF10.AT1 and fluvastatin resistant MCF10.DCIS cells with or without fluvastatin treatment. *p < 0.05

Using the MCF10A cell line model of breast cancer progression, we first examined for the deregulation of the cholesterol biosynthesis pathway during the preneoplastic stages of tumor development. This gene panel of inherent statin resistance and response was previously derived from a window trial of statin, where the higher baseline expression of the gene panel in the breast tumor correlated with decreased statin efficacy as measured by reduction in Ki67 in the tumor after 2 weeks of treatment [2, 9]. When comparing to the statin response signature reported by Kimbung et al. [9], we found that expression of many of the genes from this published signature of inherent statin resistance (70.5%; *n* = 12 of 17 tested, Fig. 1a) increased in the continuum from normal (MCF10A.P) to precancer (MCF10.DCIS). Functionally, these gene alterations appeared to correlate with differential cell growth inhibitory effects of statin. Specifically, we found fluvastatin at 5 μM and 10 μM to preferentially inhibit the colonizing ability of preneoplastic cells (MCF10.AT1) cells to form colonies (by 72.5% and 94%, respectively) as compared to MCF10.DCIS cells (39.65% and 62.76% reduction in growth respectively, Fig. 1b), [6]. Consistent with higher inherent sensitivity to fluvastatin, preneoplastic MCF10.AT1 cells had significantly lower expression of 12 out of 17 cholesterol biosynthesis pathway genes that ranged from 76% lesser expression for TM7SF2 to 15% lower expression for FDFT1 relative to MCF10.DCIS cells (*p* ≤ 0.05, Fig. 1a and supplementary Fig. 1).

### Acquired resistance to fluvastatin is mediated by restorative upregulation of cholesterol biosynthesis pathway genes

**Fig. 2:**
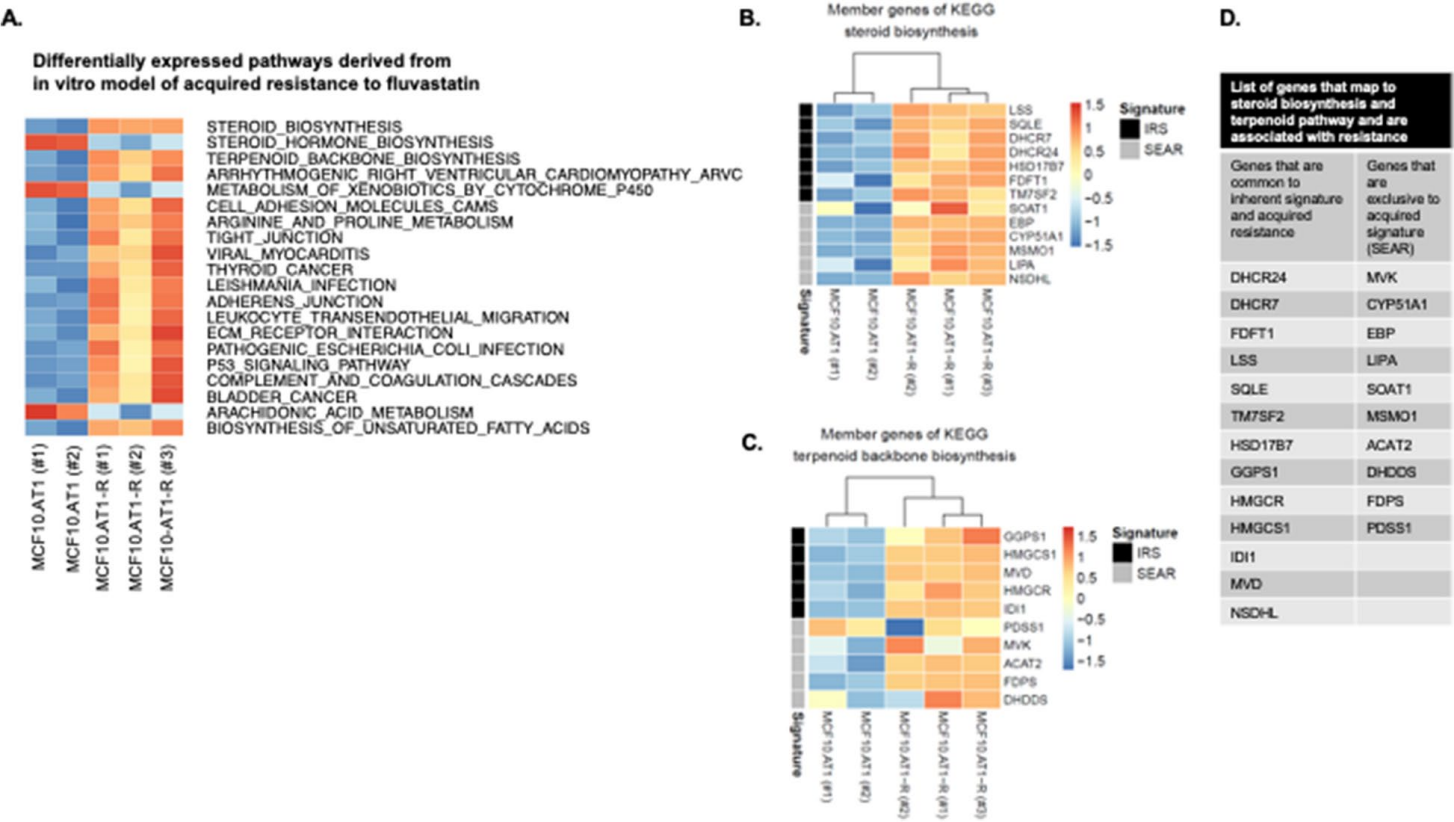
Restorative upregulation of steroid biosynthesis and terpenoid backbone biosynthesis pathways associate with fluvastatin resistance in an in vitro model of acquired resistance. **A** Heatmap showing the steroid biosynthesis and terpenoid backbone biosynthesis among the top 3 significantly upregulated pathways (FDR < 5%) and **B **& **C** the genes that map in these pathways in the fluvastatin resistant MCF10.AT-R cells**. **(**B **& **C, black bar**) The row-side color bars represent genes that are shared between steroid biosynthesis pathway and inherent gene signature (IRS) and between terpenoid backbone biosynthesis pathways and IRS. (**B **& **C, gray bar**) genes that were found to be upregulated exclusively in the MCF10.AT-R cells (SEAR). (**D**) Lists of genes that are either shared with IRS or the ones that were found to be upregulated exclusively in the MCF10.AT-R cells (SEAR) representing the unique gene signature of acquired resistance to fluvastatin

Given that resistance to statin chemoprevention may be either inherent or acquired, we next sought to determine whether the gene signatures of acquired resistance to fluvastatin were similar to those noted for intrinsic resistance, we generated a fluvastatin resistant cell line. MCF10.AT1-R was generated by exposing fluvastatin sensitive MCF10.AT1 cells to gradually increasing concentrations of fluvastatin, as described before [6]. The MCF10.AT1-R cells were 4 times more resistant to fluvastatin (IC50 = 8.5 μM) than MCF10.AT1 counterparts (IC50 = 2.1 μM as evaluated previously by MTT assay [6]). Clariom D based transcriptome assay was performed using the total cellular mRNA from three biological replicates of these cell lines to obtain gene signatures of acquired fluvastatin resistance. After ensuring data quality by principal component analysis (supplementary Fig. 2), LIMMA analysis comparing the sensitive parental line with derived resistant cell lines was performed. This revealed that 1049 probe IDs representing 913 genes were significantly altered in the resistant cells (FDR < 5%, supplementary Fig. 3). Interestingly, similar to the inherent signature [9], pathway analysis showed steroid biosynthesis, steroid hormone biosynthesis, and terpenoid backbone biosynthesis as top three significantly deregulated pathways (Fig. 2a). Heat maps of the 2 upregulated pathways- steroid biosynthesis pathway genes (*n* = 13) and the terpenoid backbone biosynthesis pathway genes (*n* = 10) are shown in the Fig. 2b and c respectively. Of the 20 genes reported in the published statin signature (called here as inherent resistance signature (IRS) [9], 13 (65%) were also found to be upregulated in the acquired resistance to fluvastatin in MCF10.AT1-R cells (Fig. 2d). In addition, a 10 gene signature exclusive to acquired resistance (SEAR) to statin was also identified. These results suggest that the gene signature that confers inherent resistance to statins is also activated/ implicated in cells that have acquired resistance to statins.

### Upregulation of steroid- and terpenoid backbone- biosynthesis pathway genes associates with fluvastatin resistance in mouse model of breast cancer

**Fig. 3:**
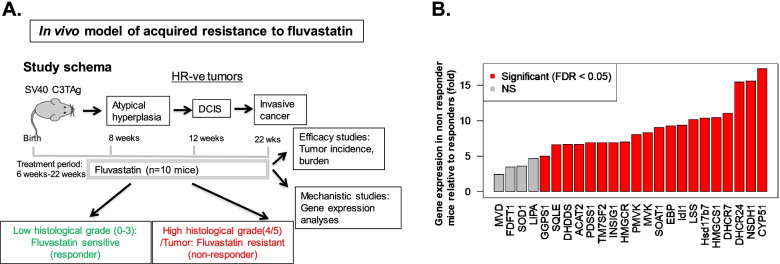
Steroid- and terpenoid backbone- biosynthesis pathway genes associate with fluvastatin resistance in a transgenic mouse model of breast cancer. **A** Schematic showing SV40C3 TAg mice treatment group, drug dosing and timeline. Fluvastatin treatment (10 mg/kg/d) was started at about 6 weeks of age and continued till 22 weeks of age through their drinking water. At the end of study, mice were sacrificed, and their mammary glands were collected and processed for RNA extraction, qPCR and histological processing. **B** Bar diagrams show qPCR validation where over expression of genes (that are part of an inherent and acquired resistance signature panel) correlates tumor outcome in fluvastatin treated SV40C3TAg mice. The Y axis depicts the fold changes of average gene expression that was calculated by using the ΔΔCt method (as described previously [14] after normalizing with ribosomal protein L19. Red color in bar diagram represents the fold changes were significant and gray represents non-significant (NS) changes at an FDR of < 5%

We next investigated the association of gene signature of acquired resistance to fluvastatin and published gene signature of inherent resistance to statin with fluvastatin efficacy in SV40 C3TAg mice. These analyses were limited to studying the expression of 16 of the 20 IRS genes and 8 of the 10 SEAR genes for which mouse homologs existed or for which specific primers could be designed (Fig. 3a). Mice were treated with either fluvastatin or vehicle starting at the age of 5–6 weeks through the age of 22 weeks and mammary gland harvested for analysis of tumor burden as described in the methods. The expression of statin response genes was compared between the tumor bearing and non-tumor bearing glands. Gene expression analysis revealed a significant (*p* ≤ 0.05) upregulation in tumor bearing glands of genes that map to steroid and terpenoid /isoprenoid backbone biosynthesis pathway genes (20 out of 24 [83.3%]) (Fig. 3b). In particular, 14 genes out of the 16 tested (87.5%) from the IRS panel were upregulated in tumor bearing mammary glands and 6 out of 8 tested (75%) SEAR genes were upregulated (Fig. 3b) in tumor bearing glands as compared to non-tumor bearing glands.

**Fig. 4:**
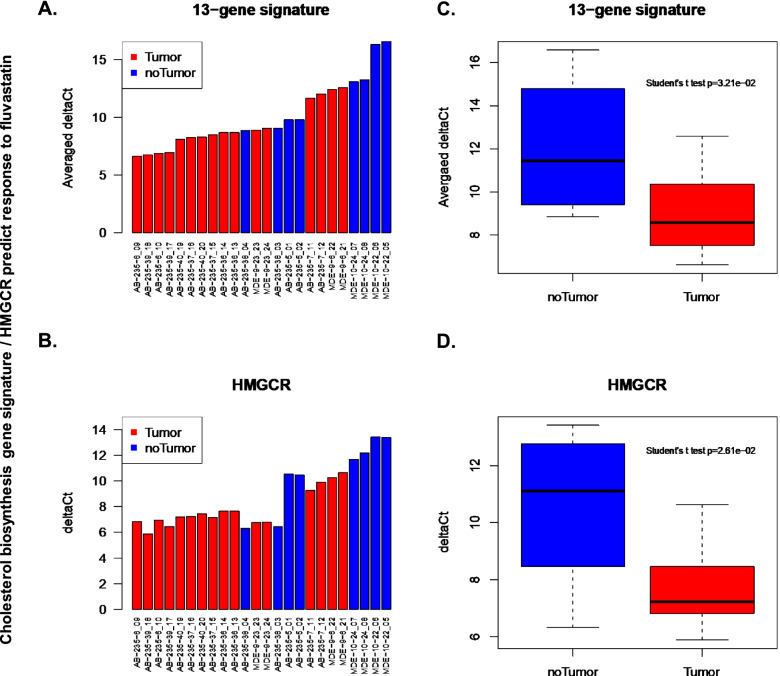
A 13-gene signature from steroid- and terpenoid backbone- biosynthesis pathways or HMGCR alone predicts fluvastatin efficacy in a transgenic mouse model of breast cancer. **A **and **B** 13-gene panel (DHCR24, DHCR7, LSS, SQLE, TM7SF2, HSD17B7, GGPS1, HMGCR, HMGCS1, IDI1, NSDHL, pMVK, INSIG1) or HMGCR alone are significantly correlated with the response to statin treatment in mice. The 13 genes of the signature were identified in both our study as well as previously published [9]. The Y-axes represent the delta Ct values that are the differences between the raw Ct values of targets and the raw Ct values of internal control ribosomal protein L19. The larger the delta Ct, the lower the expression of gene. **C **and **D** Low expression of the 13-gene panel or HMGCR alone are significantly associated with the inhibition of tumor growth in mice following the fluvastatin treatment (Student’s t-test *p* < 0.05)

We next investigated whether HMGCR alone, or a panel of genes associated with inherent statin resistance, could predict the efficacy of fluvastatin in vivo. For the panel, we selected 13 genes from the IRS that were consistently upregulated in all the model systems tested. We found that high expression of 13 gene panel from the IRS panel as well as HMGCR alone were significantly associated with the breast tumor development in fluvastatin treated mice (Fig. 4). This is in line with prior report where HMGCR was validated and found to be more predictive than the multi-gene signature of statin response as measured by reduction in Ki67 in patient breast tissue [9]. No other gene alone was found to be predictive of statin response in the prior study and thus was not validated here [9].

## Discussion

In the present study, we report that a previously published signature of inherent resistance to statin treatment in breast cancer [9] is also relevant in the preneoplastic setting as the genes in this published signature were also upregulated in the multi-step progression to invasive disease. This signature also appears to be associated with both inherent as well as acquired resistance to fluvastatin prevention therapy and largely reflect changes in the underlying steroid biosynthesis pathway. Our findings underscore the alterations that occur in the steroid biosynthesis pathway during the multi-step progression to invasive breast carcinoma and present opportunity for tailored use of statin for breast cancer chemoprevention.

The cholesterol biosynthesis pathway appears to play an important role in breast tumorigenesis. Several lines of evidence support a central role of cholesterol biosynthesis pathway in the development, progression and prognosis of breast cancer. First, HMGCR and cholesterol are both known to cause transformation of mammary cells [8, 19]. Second, high risk breast lesions, such as atypia, are known to have high levels of cholesterol in breast fluid [20]. Third, high cholesterol levels lead to an immunosuppressive environment in the breast through production of its metabolite 27-hydroxy cholesterol and promote breast cancer metastasis [21]. Fourth, elevated cholesterol is strongly associated with breast cancer reoccurrence and survival [22]. Fifth, high cholesterol biosynthesis pathway genes and HMGCR associate with poor prognosis of breast cancer patients [9]. Lastly, resistance to aromatase inhibitor therapy is mediated via epigenetic reprogramming to upregulate cholesterol biosynthesis pathway and 27-hydroxy cholesterol that eventually promote cellular invasion and in turn cause the constitutive activation of ER alpha leading to cancer cells that are refractory to therapies [23]. Additional evidence for the role of aberrant cholesterol biosynthesis pathway in breast cancer development is evident through the link between inactivation of p53- the most commonly mutated gene in the triple negative breast cancer in patients- and its ability to activate the cholesterol biosynthesis pathway [24].

In line with these observations of the relevance of the cholesterol biosynthesis pathway in breast cancer, our data demonstrates that the cholesterol biosynthesis pathway is increasingly upregulated during the multi-step progression to cancer. This implies that the window for efficacy of statin for breast cancer prevention is narrowed if initiated during late stages of progression to invasive cancer. Indeed, in prior statin chemoprevention studies, treatment of C3TAg mice prior to the onset of atypia lesions (at 6 weeks) [5] was found to be more effective in preventing the development of breast cancer as compared to starting treatment at 3 months of age, after the onset of atypia [25].

Our data also suggests that careful patient selection will be important in order to optimize benefit of statin chemoprevention and may explain why, in unselected populations, efficacy of statin chemoprevention has not been consistently observed [26]. Identifying women who at baseline have upregulation of the cholesterol biosynthesis pathway, or who develop pathway upregulation in response to therapy, will be important to ensure that treatment is avoided in such women and targeted instead to at risk populations most likely to respond. Our findings of activation of cholesterol pathway genes in histologic states further advanced along the multi-stage progression to invasive cancer (e.g., DCIS compared to atypia) additionally suggest that statin chemoprevention should be preferentially targeted to women with breast histologic findings that place them at an early stage in the multi-step tumorigenesis cascade. For populations identified as not likely to respond, co-targeting genes involved in the feedback restoration of the cholesterol pathway (e.g., HMGCS1, GGPS1 and MVK) may provide an avenue to sensitize cells resistant to statins and will be the focus of our future studies.

While our present study was conducted in an animal model and used fluvastatin, one of many available statins, we believe these findings are more generalizable. Our use of fluvastatin- a hydrophilic statin-was based on the earlier reports of its efficacy in preclinical studies [7]. We found our gene signatures of response to fluvastatin was largely similar to a gene signature of response to atorvastatin, with more than 70% of the genes shared between the two [9]. This suggests that regardless of which statin is used for chemoprevention, the same signature can be used to monitor for efficacy. In addition, although the models we used in this study are representative of basal like breast cancer, our data are similar to the findings of studies using non basal like models and patient samples [9, 27]. This observation would suggest the utility of this multigene statin response signature in all subtypes of breast cancer [9, 27].

## Conclusions

In summary, our data validates the relevance of a previously published signature of statin response in breast cancer to the preneoplastic setting and provides a gene signature that associates with responsiveness to fluvastatin for prevention of breast cancer. Similar to the cancer therapeutic domain, our work suggests that the effectiveness of targeted therapy for prevention is maximized when treatment is timed/targeted to the preneoplastic stage based on the levels of underlying target/ pathway. Collectively, the ability to identify histologic/molecular windows within the lengthy multi-step progression to invasive cancer where transformed breast cells are most responsive to statin, and the ability to further refine likelihood of statin benefit through the application of response signatures, will provide the tools to test statin as a chemoprevention agent in a highly personalized way.

## Supplementary Information

**Additional file1 : Suppl Fig. 1. **QPCR validation of a gene panel thatconstitutes inherent statin resistance signature in MCF10A breast cancer progression panel. (A, B, C).Bar diagrams show the upregulation in the cholesterol biosynthesis pathway genes in the fluvastatin resistant MCF10A.DCIS cell line relative tosensitive MCF10.AT1 cells as revealed by qPCR. The Y axis depicts mRNAexpression in fold after normalizing with ribosomal protein L19 using ΔΔCt method. Values represent mean +/- SEM *p<0.05. **Suppl Fig. 2.** Principal component analysis (PCA) was performed on the expression levels of all genes to estimate the correlation between samples. The first principal component that explains 98.1% variation distinctly separates the sample A03 from the rest. This variation cannot be explained biologically and is most likely due to the systematic variations. Therefore, sample A03 was removed from downstream genomic analysis. **Suppl Fig. 3**. Genes that significantly change between fluvastatin sensitive MCF10.AT1 and resistant MCF10.AT1-R cells. (A) Heatmap showing expression pattern of 913 genes that significantly change between fluvastatin sensitive MCF10.AT1 and resistant MCF10.AT1-R cells derived by taking log2 transformed gene expression values in this two-way unsupervised heat map. (FDR<5%). **Suppl Fig. 4.** Upregulation in the expression of genes that map to the steroid- and terpenoid backbone- biosynthesis pathway in non-responder mice mammary tissues. Box plots shows qPCR validation that shows that over expression of genes (that are part of an inherent and acquired resistance signature panel) as depicted by lower Ct values correlates with tumor outcome in fluvastatin treated SV40 C3TAg mice. The Y axis depicts the fold changes of average gene expression that was calculated by using the ΔΔCt method after normalizing with ribosomal protein L19. A FDR adjusted p value< 0.05 was considered as significant change.

## Data Availability

Datasets generated during the current study are available in the GEO repository and can be accessed through accession # GSE174673 and the web link: https://www.ncbi.nlm.nih.gov/geo/query/acc.cgi?acc=GSE174673.

## Abbreviations

AT1: Atypia
DCIS: Ductal carcinoma in situ
TNBC: Triple negative breast cancer
ER: Estrogen receptor
EGF: Epidermal growth factor
IC 50: Concentration that inhibits 50% cell proliferation
FDR: False discovery rate
MSigDB: Molecular signature database
IRS: Inherent resistance gene signature
SEAR: Gene signature exclusive to acquired resistance
HMGCR: HMG CoA reductase
HMGCS1: HMG CoA synthase 1
MVA: Mevalonic acid
MVD: Mevalonate Diphosphate Decarboxylase
MVK: Mevalonate kinase
GGPS1: Geranylgeranyl Diphosphate Synthase 1
