# Supplementary material for: Gene signature associated with resistance to fluvastatin chemoprevention for breast cancer
Source: BMC Cancer. 2022 Mar 17;22:282. doi: 10.1186/s12885-022-09353-2 (PMC8928625; doi:10.1186/s12885-022-09353-2)
Supplement: Supplementary file 1 — Additional file1 : Suppl Fig. 1. QPCR validation of a gene panel thatconstitutes inherent statin resistance signature in MCF10A breast cancer progression panel. (A, B, C).Bar diagrams show the upregulation in the cholesterol biosynthesis pathway genes in the fluvastatin resistant MCF10A.DCIS cell line relative tosensitive MCF10.AT1 cells as revealed by qPCR. The Y axis depicts mRNAexpression in fold after normalizing with ribosomal protein L19 using ΔΔCt method. Values represent mean +/- SEM *p<0.05. Suppl Fig. 2. Principal component analysis (PCA) was performed on the expression levels of all genes to estimate the correlation between samples. The first principal component that explains 98.1% variation distinctly separates the sample A03 from the rest. This variation cannot be explained biologically and is most likely due to the systematic variations. Therefore, sample A03 was removed from downstream genomic analysis. Suppl Fig. 3. Genes that significantly change between fluvastatin sensitive MCF10.AT1 and resistant MCF10.AT1-R cells. (A) Heatmap showing expression pattern of 913 genes that significantly change between fluvastatin sensitive MCF10.AT1 and resistant MCF10.AT1-R cells derived by taking log2 transformed gene expression values in this two-way unsupervised heat map. (FDR<5%). Suppl Fig. 4. Upregulation in the expression of genes that map to the steroid- and terpenoid backbone- biosynthesis pathway in non-responder mice mammary tissues. Box plots shows qPCR validation that shows that over expression of genes (that are part of an inherent and acquired resistance signature panel) as depicted by lower Ct values correlates with tumor outcome in fluvastatin treated SV40 C3TAg mice. The Y axis depicts the fold changes of average gene expression that was calculated by using the ΔΔCt method after normalizing with ribosomal protein L19. A FDR adjusted p value< 0.05 was considered as significant change. [file 12885_2022_9353_MOESM1_ESM.pptx]

## Slide 1
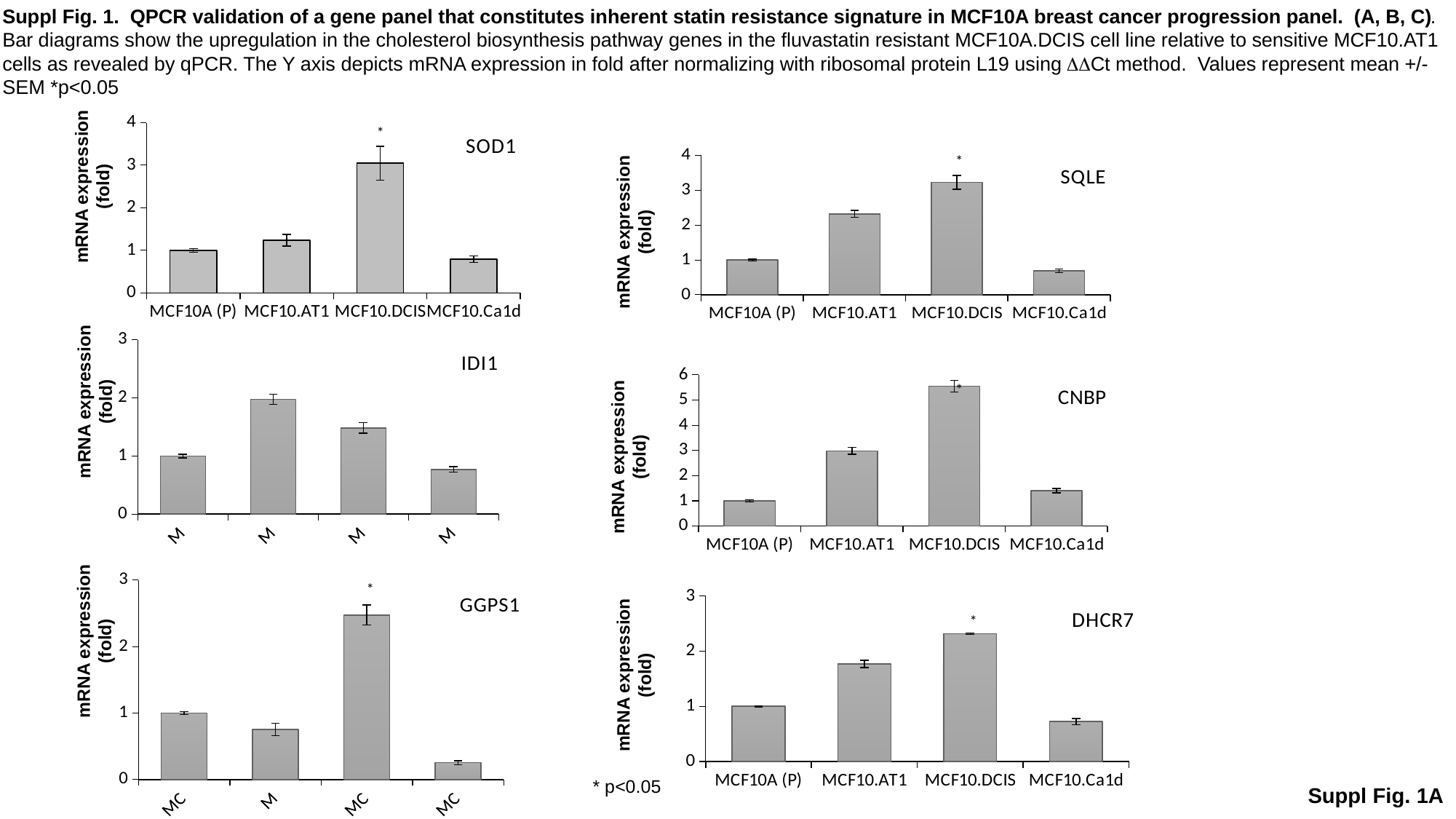

Suppl Fig. 1. QPCR validation of a gene panel that constitutes inherent statin resistance signature in MCF10A breast cancer progression panel. (A, B, C). Bar diagrams show the upregulation in the cholesterol biosynthesis pathway genes in the fluvastatin resistant MCF10A.DCIS cell line relative to sensitive MCF10.AT1 cells as revealed by qPCR. The Y axis depicts mRNA expression in fold after normalizing with ribosomal protein L19 using ΔΔCt method. Values represent mean +/- SEM *p<0.05
### Chart: SOD1
| Category | SOD1 |
|---|---|
| MCF10A (P) | 1.0 |
| MCF10.AT1 | 1.237590086772221 |
| MCF10.DCIS | 3.047318573065061 |
| MCF10.Ca1d | 0.793581531362148 |*
*
### Chart: SQLE
| Category | SQLE |
|---|---|
| MCF10A (P) | 1.0 |
| MCF10.AT1 | 2.319470389958294 |
| MCF10.DCIS | 3.223569908939438 |
| MCF10.Ca1d | 0.688289684239324 |mRNA expression (fold)
mRNA expression (fold)
### Chart: IDI1
| Category | IDI1 |
|---|---|
| MCF10A (P) | 1.0 |
| MCF10.AT1 | 1.973710065694109 |
| MCF10.DCIS | 1.482556897562238 |
| MCF10.Ca1d | 0.766590303047487 |
### Chart: CNBP
| Category | CNBP |
|---|---|
| MCF10A (P) | 1.0 |
| MCF10.AT1 | 2.982014311140954 |
| MCF10.DCIS | 5.550890353098408 |
| MCF10.Ca1d | 1.402704988511548 |*
mRNA expression (fold)
mRNA expression (fold)
### Chart: GGPS1
| Category | GGPS1 |
|---|---|
| MCF10A (P) | 1.0 |
| MCF10.AT1 | 0.750889703659542 |
| MCF10.DCIS | 2.472340438395606 |
| MCF10.Ca1d | 0.254226765546662 |*
### Chart: DHCR7
| Category | DHCR7 |
|---|---|
| MCF10A (P) | 1.0 |
| MCF10.AT1 | 1.76628329843875 |
| MCF10.DCIS | 2.317946804664626 |
| MCF10.Ca1d | 0.721710893437566 |*
mRNA expression (fold)
mRNA expression (fold)
* p<0.05
Suppl Fig. 1A

## Slide 2
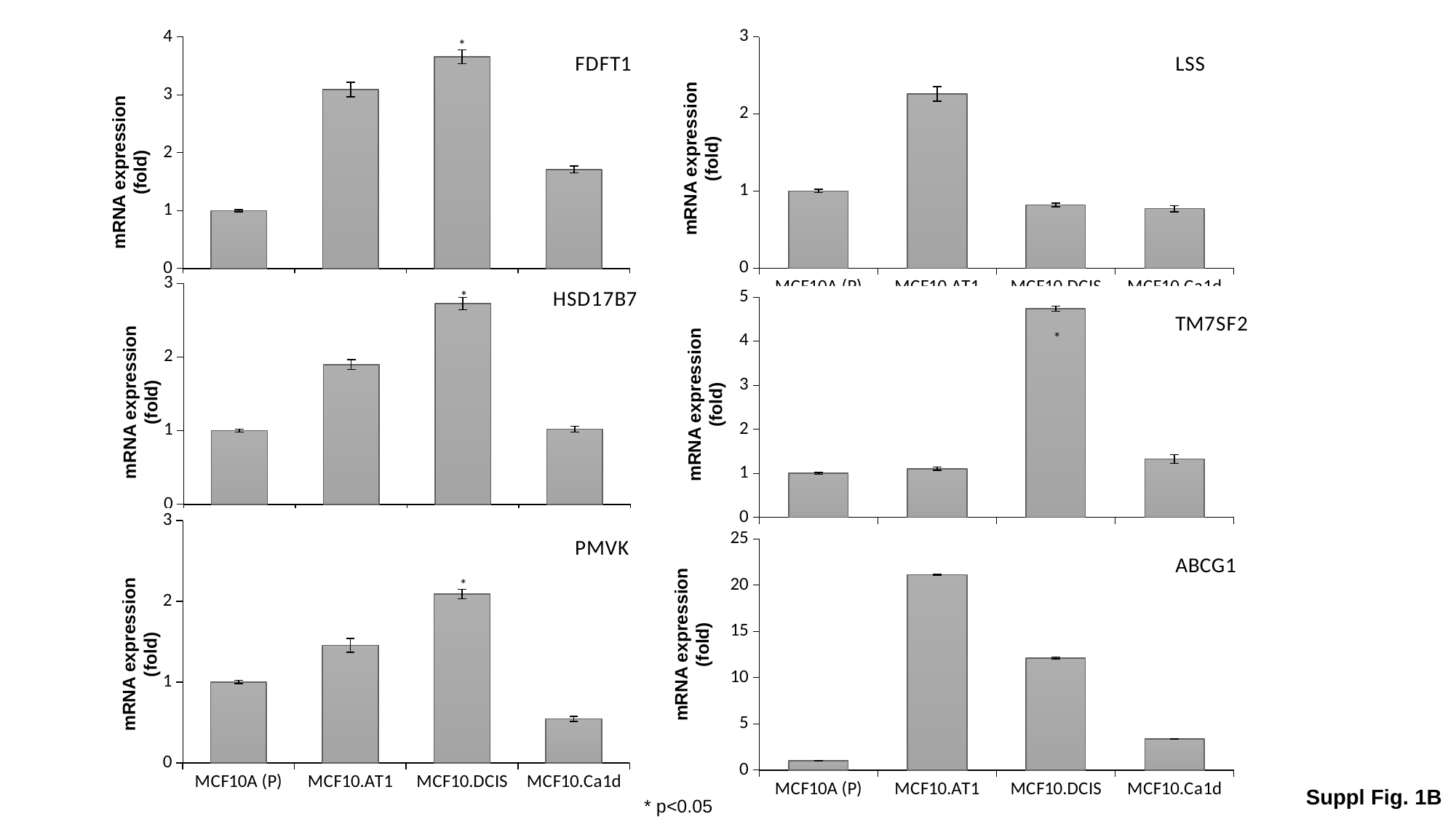

### Chart: LSS
| Category | LSS |
|---|---|
| MCF10A (P) | 1.0 |
| MCF10.AT1 | 2.26028401711042 |
| MCF10.DCIS | 0.81914790897534 |
| MCF10.Ca1d | 0.76989893828285 |
### Chart: FDFT1
| Category | FDFT1 |
|---|---|
| MCF10A (P) | 1.0 |
| MCF10.AT1 | 3.092035208051062 |
| MCF10.DCIS | 3.658797080855691 |
| MCF10.Ca1d | 1.709808604029513 |*
mRNA expression (fold)
mRNA expression (fold)
### Chart: HSD17B7
| Category | HSD17B7 |
|---|---|
| MCF10A (P) | 1.0 |
| MCF10.AT1 | 1.895277381040281 |
| MCF10.DCIS | 2.724108195581334 |
| MCF10.Ca1d | 1.020688445905473 |*
### Chart: TM7SF2
| Category | TM7SF2 |
|---|---|
| MCF10A (P) | 1.0 |
| MCF10.AT1 | 1.103220404135447 |
| MCF10.DCIS | 4.73918164578854 |
| MCF10.Ca1d | 1.326326254033489 |*
mRNA expression (fold)
mRNA expression (fold)
### Chart: PMVK
| Category | PMVK |
|---|---|
| MCF10A (P) | 1.0 |
| MCF10.AT1 | 1.454153008046392 |
| MCF10.DCIS | 2.088889105023985 |
| MCF10.Ca1d | 0.545613904912095 |
### Chart: ABCG1
| Category | ABCG1 |
|---|---|
| MCF10A (P) | 1.0 |
| MCF10.AT1 | 21.14169838662728 |
| MCF10.DCIS | 12.12032588139936 |
| MCF10.Ca1d | 3.36052636184639 |*
mRNA expression (fold)
mRNA expression (fold)
Suppl Fig. 1B
* p<0.05

## Slide 3
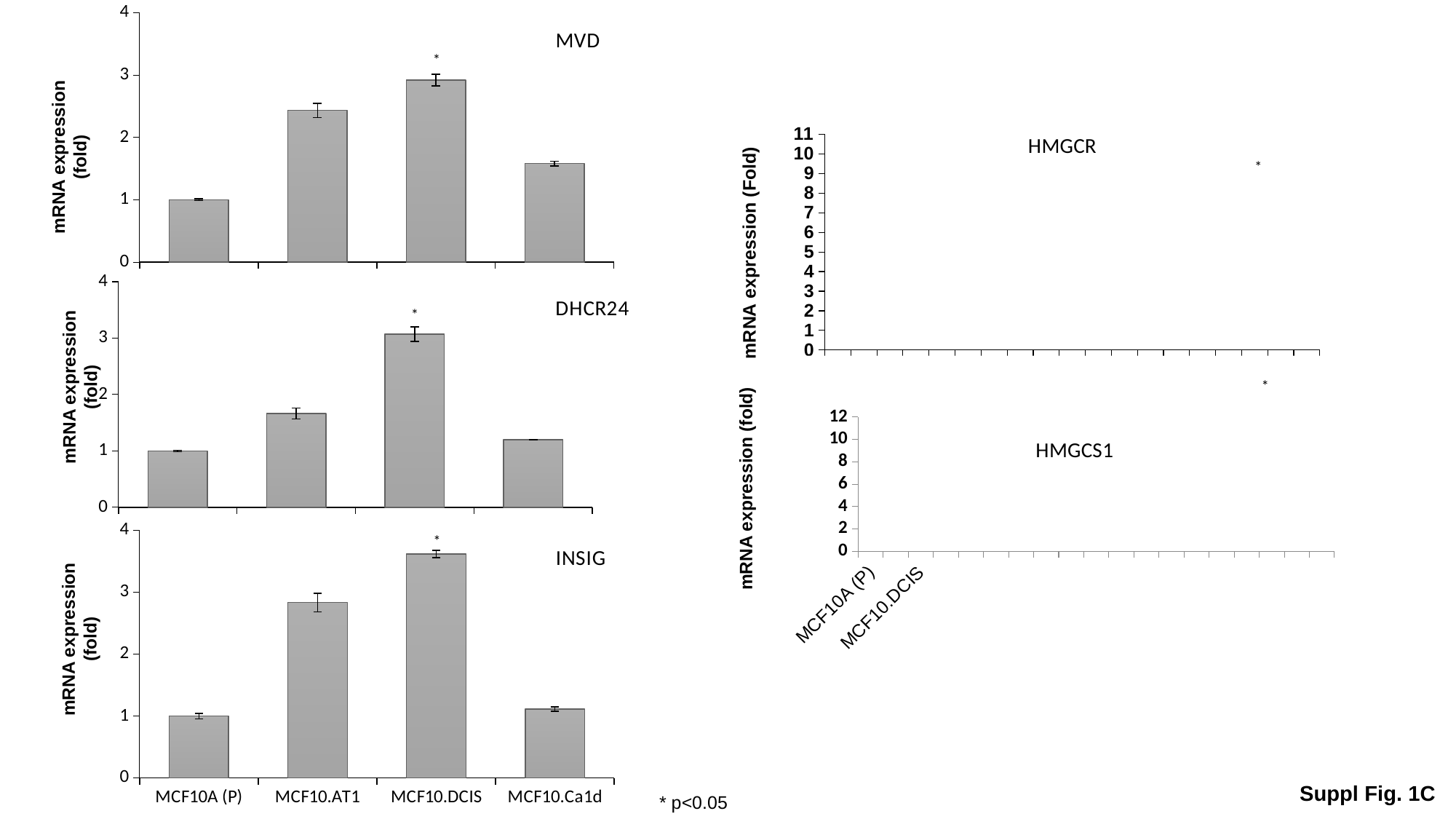

### Chart: MVD
| Category | MVD |
|---|---|
| MCF10A (P) | 1.0 |
| MCF10.AT1 | 2.433525047195362 |
| MCF10.DCIS | 2.920828624514026 |
| MCF10.Ca1d | 1.580168379984481 |*
### Chart: HMGCR
| Category | |
|---|---|
| 10A (P) | 1.0 |
| AT1 | 1.5 |
| DCIS | 1.7 |
| Ca1d | 2.46 |mRNA expression (fold)
*
mRNA expression (Fold)
### Chart: DHCR24
| Category | DHCR24 |
|---|---|
| MCF10A (P) | 1.0 |
| MCF10.AT1 | 1.663546257244425 |
| MCF10.DCIS | 3.070759161660341 |
| MCF10.Ca1d | 1.20006856747423 |*
### Chart: HMGCS1
| Category | |
|---|---|
| MCF10A (P) | 1.0 |
| MCF10.AT1 | 3.18 |
| MCF10.DCIS | 2.1 |
| MCF10.CA1D | 5.5 |mRNA expression (fold)
*
mRNA expression (fold)
### Chart: INSIG
| Category | INSIG |
|---|---|
| MCF10A (P) | 1.0 |
| MCF10.AT1 | 2.832753276478276 |
| MCF10.DCIS | 3.615520241558062 |
| MCF10.Ca1d | 1.10999616946327 |*
mRNA expression (fold)
Suppl Fig. 1C
* p<0.05

## Slide 4
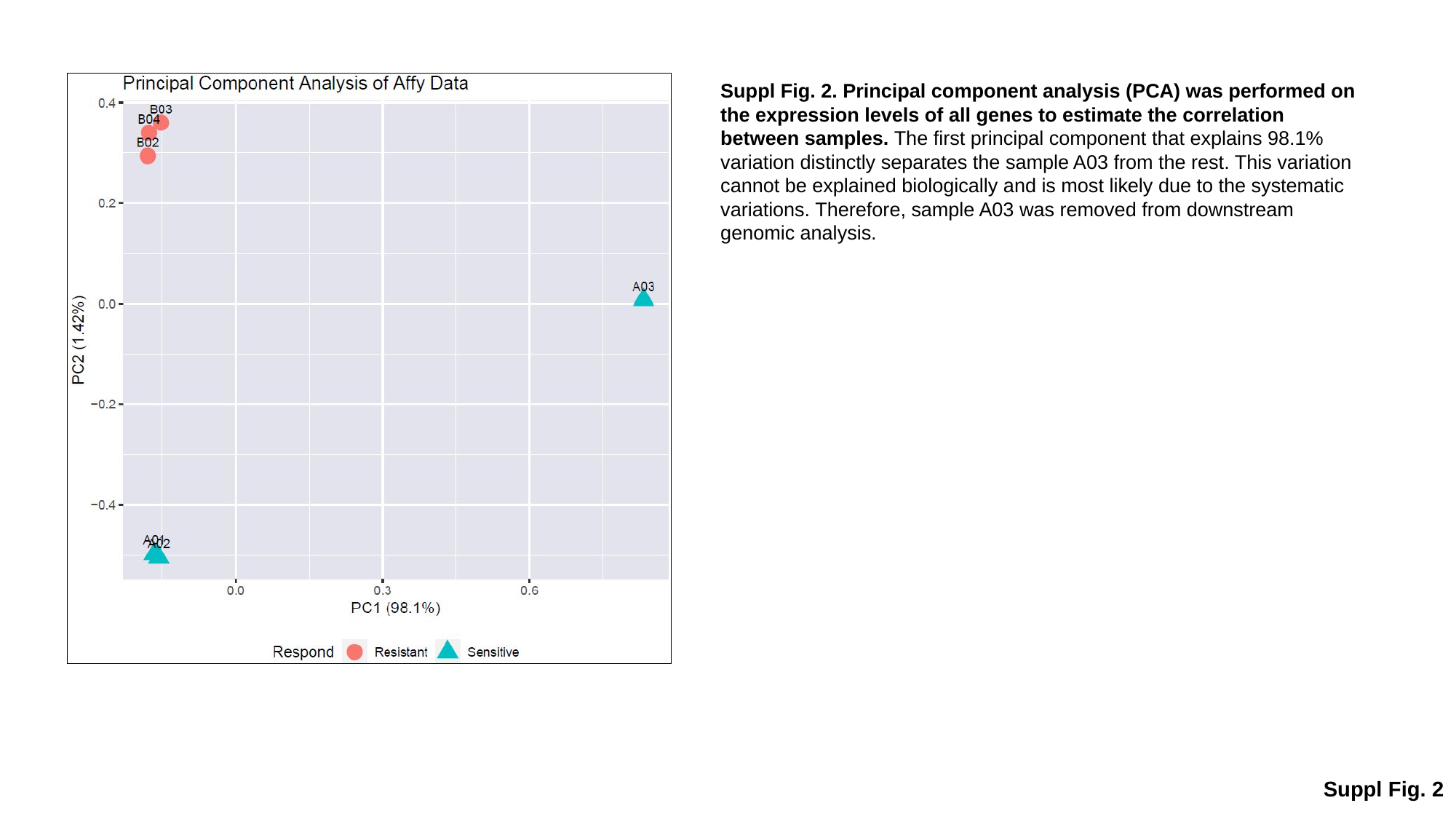

Suppl Fig. 2. Principal component analysis (PCA) was performed on the expression levels of all genes to estimate the correlation between samples. The first principal component that explains 98.1% variation distinctly separates the sample A03 from the rest. This variation cannot be explained biologically and is most likely due to the systematic variations. Therefore, sample A03 was removed from downstream genomic analysis.
Suppl Fig. 2

## Slide 5
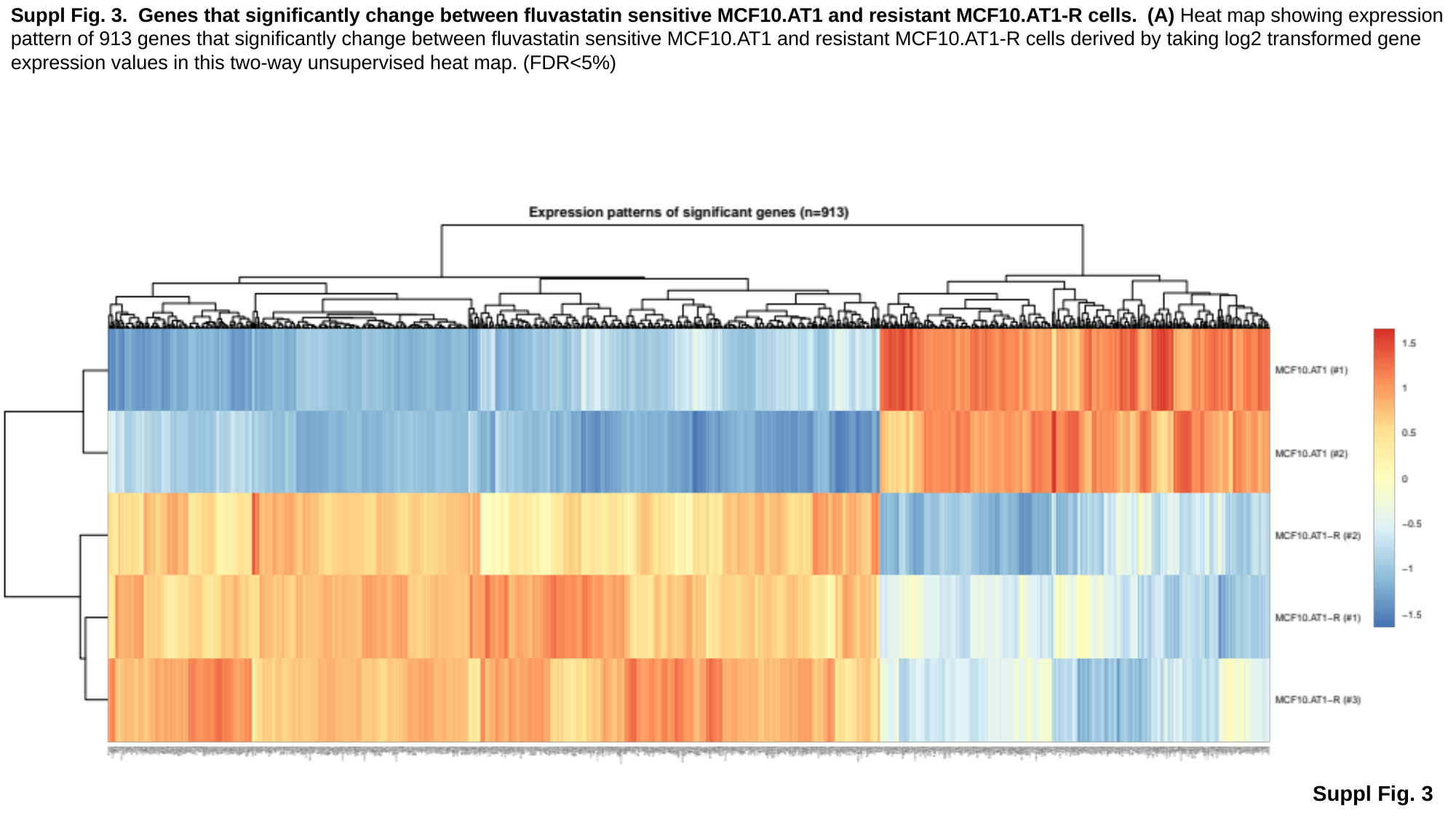

Suppl Fig. 3. Genes that significantly change between fluvastatin sensitive MCF10.AT1 and resistant MCF10.AT1-R cells. (A) Heat map showing expression pattern of 913 genes that significantly change between fluvastatin sensitive MCF10.AT1 and resistant MCF10.AT1-R cells derived by taking log2 transformed gene expression values in this two-way unsupervised heat map. (FDR<5%)
Suppl Fig. 3

## Slide 6
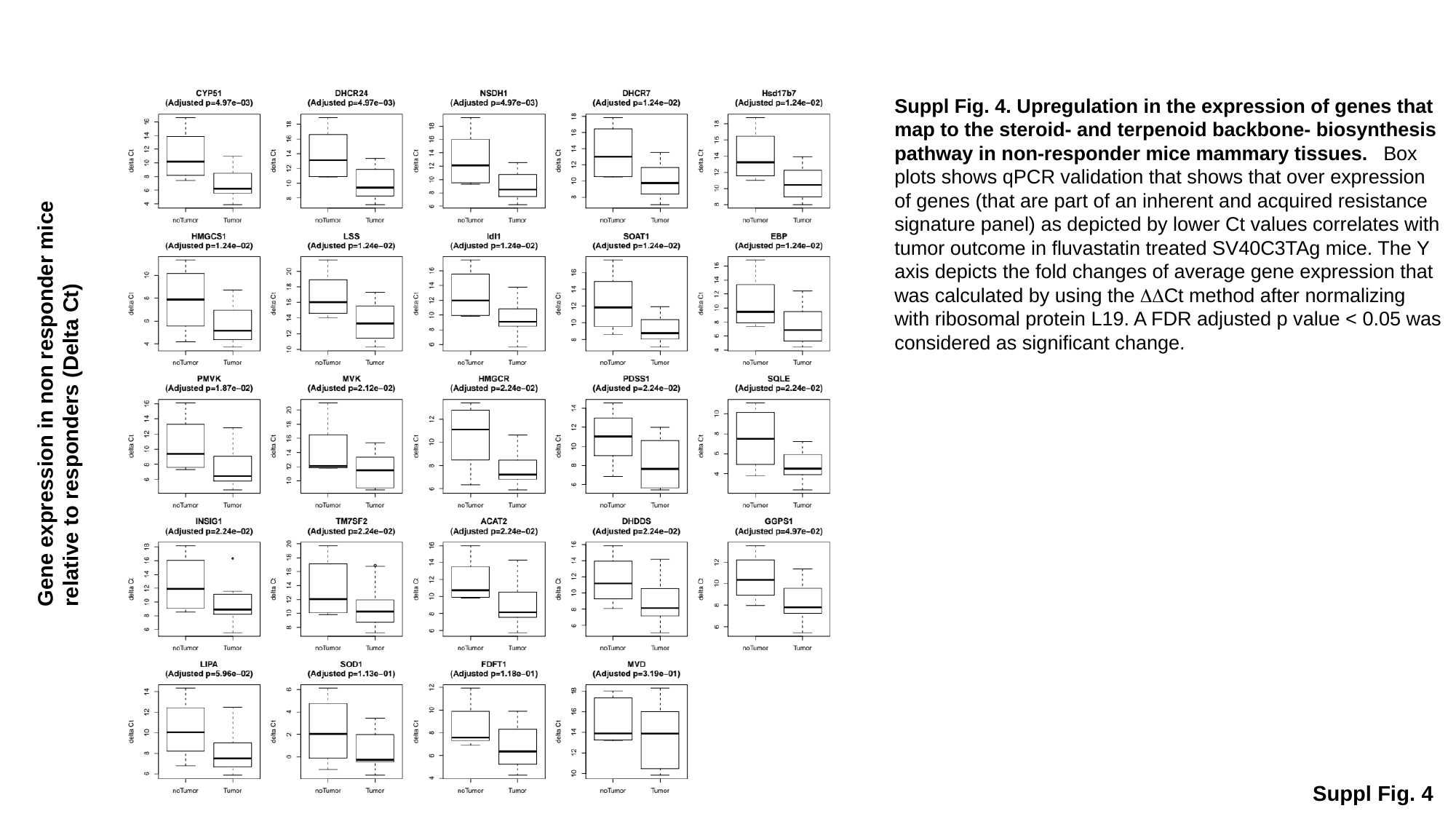

Suppl Fig. 4. Upregulation in the expression of genes that map to the steroid- and terpenoid backbone- biosynthesis pathway in non-responder mice mammary tissues. Box plots shows qPCR validation that shows that over expression of genes (that are part of an inherent and acquired resistance signature panel) as depicted by lower Ct values correlates with tumor outcome in fluvastatin treated SV40C3TAg mice. The Y axis depicts the fold changes of average gene expression that was calculated by using the ΔΔCt method after normalizing with ribosomal protein L19. A FDR adjusted p value < 0.05 was considered as significant change.
Gene expression in non responder mice
relative to responders (Delta Ct)
Suppl Fig. 4
